# Assessing Lighting Quality and Occupational Outcomes in Intensive Care Units: A Case Study from the Democratic Republic of Congo

**DOI:** 10.3390/ijerph22101511

**Published:** 2025-10-01

**Authors:** Jean-Paul Kapuya Bulaba Nyembwe, John Omomoluwa Ogundiran, Nsenda Lukumwena, Hicham Mastouri, Manuel Gameiro da Silva

**Affiliations:** 1 Department of Mechanical Engineering, ADAI, University of Coimbra, Pólo II, Rua Luís Reis Santos, 3030-788 Coimbra, Portugal; 2 Department of Architectural Route Kasapa, New Horizons University, 2465, Gambela, Lubumbashi 1280, Democratic Republic of the Congo; 3 Department of Civil Engineering, University Official of Mbuji-Mayi, Av Kalonji no 27, Q/Kansele, Mbuji-Mayi 8330, Democratic Republic of the Congo; 4 School of Architecture, Planning, and Design, Mohammed VI Polytechnic University, Lot 660, Hay Moulay Rachid, Ben Guerir 43150, Morocco

**Keywords:** Democratic Republic of Congo, intensive care units, lighting quality, staff well-being, visual comfort

## Abstract

This study presents a comprehensive assessment of lighting conditions in the Intensive Care Units (ICUs) of two major hospitals in the Democratic Republic of Congo (DRC): Hospital du Cinquantenaire in Kinshasa and Jason Sendwe Hospital in Lubumbashi. A mixed-methods approach was employed, integrating continuous illuminance monitoring with structured staff surveys to evaluate visual comfort in accordance with the EN 12464-1 standard for indoor workplaces. Objective measurements revealed that more than 52.2% of the evaluated ICU workspaces failed to meet the recommended minimum illuminance level of 300 lux. Subjective responses from healthcare professionals indicated that poor lighting significantly reduced job satisfaction by 40%, lowered self-rated task performance by 30%, decreased visual comfort scores from 4.1 to 2.6 (on a 1–5 scale), and increased the prevalence of well-being symptoms (eye fatigue, headaches) by 25–35%. Frequent complaints included eye strain, glare, and discomfort with posture, with these issues often exacerbated during the rainy season due to reduced natural daylight. The study highlights critical deficiencies in current lighting infrastructure and emphasizes the need for urgent improvements in clinical environments. Moreover, inconsistent energy supply to these healthcare settings also impacts the assurance of visual comfort. To address these shortcomings, the study recommends transitioning to energy-efficient LED lighting, enhancing access to natural light, incorporating circadian rhythm-based lighting systems, enabling individual lighting control at workstations, and ensuring a consistent power supply via the integration of solar inverters to the grid supply. These interventions are essential not only for improving healthcare staff performance and safety but also for supporting better patient outcomes. The findings offer actionable insights for hospital administrators and policymakers in the DRC and similar low-resource settings seeking to enhance environmental quality in critical care facilities.

## 1. Introduction

Illuminance, measured in lux (lx), quantifies the luminous flux incident on a surface and is a core determinant of visual comfort in workplaces, including ICUs. Standards such as EN 12464-1 recommend a minimum illumination level of ≥300 lx for clinical tasks; however, compliance in low-resource hospital settings is largely undocumented. Moreover, lighting in healthcare is more than just a necessity; it is a vital element that can make the difference between precision and error, between comfort and strain, ultimately shaping the quality of care delivered to patients. In healthcare, the satisfaction and visual comfort of employees are pivotal factors in shaping the overall quality of healthcare delivery [1,2,3]. Beyond the conventional considerations of thermal, acoustic, and air quality comfort, the significance of visual comfort, particularly in terms of lighting conditions, cannot be overstated when it comes to the accurate execution of tasks and the provision of optimal patient care [4,5,6]. Recent research has highlighted a significant correlation between illuminated environments and job satisfaction among healthcare professionals. Subpar illuminance levels and flickering in lighting systems can lead to eye strain, discomfort, glare, and fatigue, negatively impacting overall well-being [7]. These issues can also disrupt sleep patterns and compromise safety standards within healthcare facilities.

In hospitals, lighting is a critical aspect of facility design and must be tailored to the specific activities conducted in various areas [8]. However, achieving optimal lighting conditions remains challenging. Hospital lighting is often reported as suboptimal, with illuminance levels ranging from 50 to 300 lux, which can harm patients’ well-being during their hospital stays [9]. Enhancing visual comfort through appropriate facade selection, retrofitting ward lighting, and improving color temperature has been shown to benefit both patient and staff comfort significantly [10,11]. Poor lighting may make workers uncomfortable and impair their visual and overall performance [12]. Eyestrain is one of the negative health impacts that may result from inadequate lighting, discomforting glare, and flickering light [13]. Eyestrain can lead to headaches, loss of eyesight, and other health issues [12]. Optimal performance, on the other hand, occurs within a specific physical environment [14]. Indirect ambient light and direct, adjustable work light provide the best comfort, health, and productivity advantages in office lighting systems [15]. Additionally, caregivers and employees emphasize the need for more windows to enhance the benefits of natural light on physical and emotional health, highlighting the importance of incorporating humanization into hospital design [16]. Recent studies emphasize the critical role of lighting in ICU environments. Dynamic lighting systems have been shown to promote circadian alignment and support delirium prevention protocols [17]. Nighttime lighting disruption is common in ICUs, adversely affecting patient sleep quality and circadian health [18]. Nighttime ambient light levels have also been identified as significant predictors of delirium risk in ICU patients [19]. Multidisciplinary research highlights the interplay between lighting and other indoor environmental quality factors, such as noise and thermal conditions, in shaping patient outcomes [20]. Artificial lighting is essential in hospitals, as healthcare professionals, such as nurses and doctors, require consistent and reliable lighting to carry out their duties, regardless of the natural light conditions [21]. However, artificial lighting, especially at night, can negatively impact patients by disrupting sleep patterns, potentially leading to various health issues such as diabetes, heart disease, and mood disorders [22,23]. Hospital administrators and designers face a complex challenge in balancing the need for appropriate lighting for healthcare professionals while minimizing disruptions to patients’ rest and circadian rhythms. Despite the availability of international guidelines such as ISO 8995-1 [24] and World Health Organization (WHO) recommendations for healthcare lighting, these are rarely enforced in many low-income countries.

Despite this growing evidence, little research has been conducted in low-income hospital settings within Sub-Saharan Africa. Studies on surgical lighting in Ethiopia and Liberia reveal persistent ergonomic and quality challenges [25]. According to WHO, 15 percent of health facilities in the region lack electricity entirely, and only about 50 percent of hospitals have reliable access—constraints that directly affect lighting feasibility (World Health Organization). Historical reviews indicate that as few as 34 percent of hospitals in surveyed countries have reliable electricity [26], while rural electrification efforts, such as solar mini-grids, offer promising solutions [27]. Previous research in ICUs has shown that measured illuminance often falls below target levels, with reported consequences for visual comfort, task accuracy, and staff well-being [28,29,30]. In tropical hospitals, a few small-scale studies [26,31,32] have documented similar shortfalls, which are compounded by an unreliable power supply and dependence on daylight. However, these studies have rarely integrated objective light measurements with staff-reported occupational outcomes. Consideration of numerous physical environment features, including lighting levels and visual distractions, is crucial in hospital facility design [22,23]. Despite mounting evidence that the physical environment influences safety and health outcomes, many healthcare systems in sub-Saharan Africa (SSA) have not prioritized lighting as a safety intervention [23]. Moreover, there is the prevalent issue of epileptic power supply in many sub-Saharan African (SSA) countries, including the DRC [26], which hinders a steady power supply; consequently, ensuring adequate visual comfort in health settings has become a necessity. Additionally, the absence of IEQ regulations, the paucity of scientific data, and the inadequacy of sustainability policies that address the regional peculiarities of many developing tropical regions in Africa reinforce the rationale for the current study’s assessment campaign in healthcare settings [6,33,34]. To the best of the authors’ knowledge, there is a notable lack of attention to establishing proper lighting standards in healthcare facilities in the Democratic Republic of the Congo (DRC) at the time of the current study. Despite the recognized importance of adequate lighting for patient safety and staff well-being, to our knowledge, no peer-reviewed studies have quantified ICU lighting conditions in the DRC, nor examined their associations with staff perceptions and occupational outcomes. This gap is critical given the frequent reliance on daylight in tropical hospitals, power instability, and limited access to energy-efficient lighting systems.

Given the aforementioned challenges, including the scarcity of indoor environmental quality studies (IEQ) in the DRC and many SSA countries [2], this study as part of an ongoing IEQ assessment campaign, examines the impact of current lighting conditions in Intensive Care Units (ICUs) within hospitals in the DRC on the satisfaction of healthcare staff, job performance, and overall well-being. They suggest that the existing lighting conditions in these ICUs are significantly below recommended standards, contributing to lower staff satisfaction, decreased job performance, and adverse health effects, ultimately compromising the quality of patient care. This study addresses this gap by conducting continuous illuminance monitoring and a structured staff survey in ICUs in two major hospitals in the DRC, assessing compliance with EN 12464-1 [35], identifying perception–measurement mismatches, and evaluating associations between lighting conditions, visual comfort, and occupational well-being in the sub-Saharan African context. Our research defines visual comfort within ICU settings as a multifaceted concept encompassing both subjective perception and objective capabilities, such as adequate illuminance and adaptable lighting [36]. By capturing healthcare professionals’ evaluations of lighting adequacy, including light type, level, and flicker, this study offers a deeper understanding of environmental stressors that affect staff well-being [37]. Moreover, by integrating seasonal variation (dry vs. rainy seasons) and institutional setting (two hospitals), this study makes a unique contribution to the understanding of how lighting conditions impact healthcare outcomes in tropical, resource-constrained environments. The dual-method approach provides valuable evidence for improving lighting design, IEQ regulations, energy policy, and hospital safety standards in developing countries facing similar infrastructural limitations.

Notably, there are twelve hospitals in Kinshasa, although it is estimated to have a population of 17 million, while Lubumbashi has only four hospitals to serve its population of about 3.5 million people. The Hospital du Cinquantenaire (HDC) in Kinshasa and Jason Sendwe Hospital (JSH) in Lubumbashi are poised to provide a wide range of health solutions to residents of these urban, suburban communities, as well as beyond the region. This reinforces the rationale of the current study’s objective, including the select case study ICUs of public hospitals. Therefore, enhancing the adequacy of IEQ in these hospitals contributes to assuring comfort and well-being, consequently enhancing health services and reducing health burdens. Additionally, the current study findings and recommendations, being part of an ongoing IEQ assessment campaign of hospital ICUs in the DRC [6,38,39], provide a referral basis for improvement to hospital buildings in design, operation, and management, including the adoption of better regulatory practices. Moreover, the current assessment campaign raises awareness and provides scientific evidence of poor lighting in hospitals, prompting healthcare stakeholders to intervene. These interventions will ultimately impact and contribute to mitigating the associated health burdens of defective IEQ in public hospitals across the study region.

## 2. Materials and Methods

**Figure 1:**
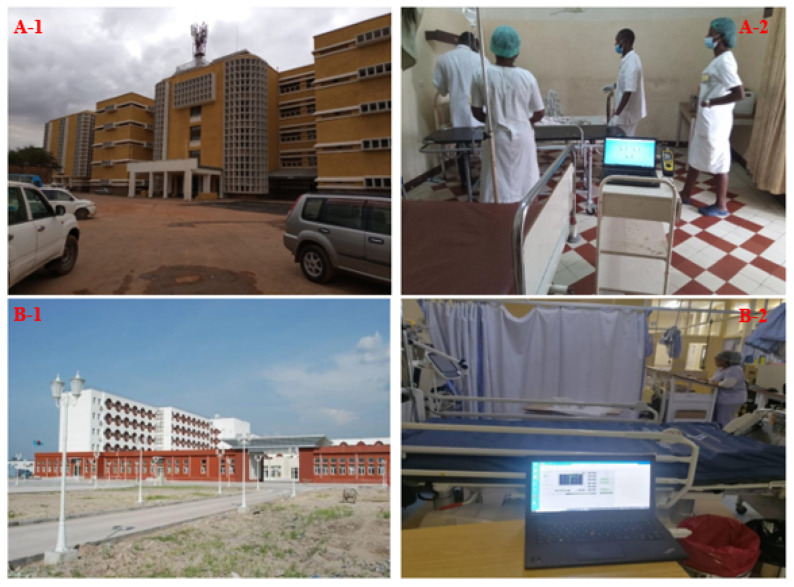
(**A-1**,**A-2**) JSH: exterior and ICU lighting measurement. (**B-1**,**B-2**) JSH: exterior and ICU lighting measurement.

**Figure 2:**
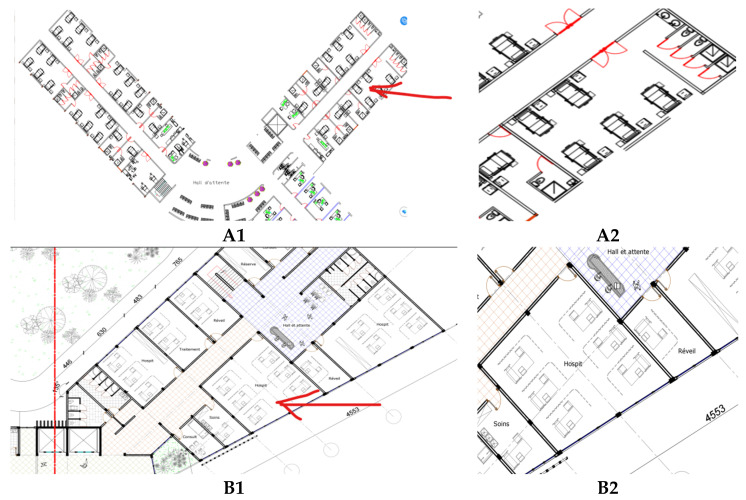
A schematic plan excerpt of the studied ICUs: (**A1**,**A2**) are for JSH, while (**B1**,**B2**) are for HDC.

The study was conducted in the ICUs of two prominent hospitals in the DRC: Hospital du Cinquantenaire (HDC) in Kinshasa and Jason Sendwe Hospital (JSH) in Lubumbashi. Established in 1924, HDC is the largest and most visited hospital in the DRC, spanning 40,000 m^2^ with seven floors above ground, housing approximately 2000 patient beds, and employing around 1200 healthcare professionals. As a national referral center located in the capital city, it plays a pivotal role in providing advanced healthcare services to a large proportion of the country’s population. Similarly, JSH, located in Lubumbashi, the DRC’s second-largest city, is a major regional healthcare provider, with an operational capacity of 240 beds and a workforce of about 500 individuals at the time of the study. It serves as a key referral and teaching hospital for the southern provinces. These two facilities were selected for their strategic importance, high patient throughput, and representative ICU environments in both the capital and a major provincial city. Their inclusion enables comparative analysis across different climatic, urban, and operational contexts, making the findings more relevant to the national healthcare system. The research focused specifically on the ICUs, where lighting quality is critical for patient safety, clinical accuracy, and staff performance. Notably, both hospitals primarily rely on fluorescent tube lighting throughout their facilities, including the ICUs. Figure 1 and Figure 2 illustrate key characteristics of the case study buildings.

Figure 1 presents the exterior and interior characteristics of the two hospitals under study. Panels A-1 and A-2 illustrate JSH, a multi-story building with a yellowish-brown façade and modern design. Its interior is characterized by red-and-white checkered flooring, light-colored walls, and fluorescent ceiling lighting, which together create a bright environment. Figure 1(B-1,B-2) shows HDC, distinguished by a predominantly white façade accented with red details and large rectangular windows. Inside, the ICU benefits from generous natural daylight, supplemented by fluorescent lighting to ensure uniform illumination for clinical tasks.

Figure 2 provides schematic plan excerpts of the studied ICUs, highlighting their spatial organization and measurement setup. Figure 2(A1, A2) depicts the JSH ICU, while Plans B1 and B2 illustrate the HDC ICU. The red arrows in the figure indicate the specific locations where the IEQ multiprobe was positioned to collect illuminance data in patient rooms and circulation areas. This integration of architectural context, interior conditions, and measurement placement ensures a comprehensive basis for analyzing the indoor environmental quality of the two hospitals.

### 2.1. Data Collection Approach

**Figure 3:**
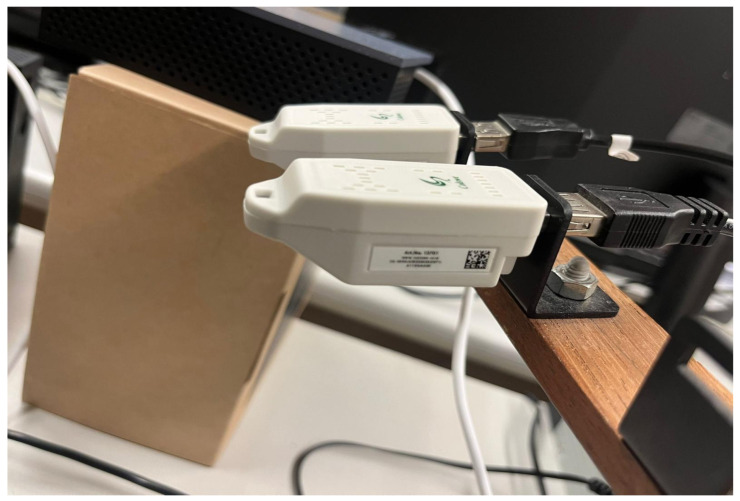
Calibration of the IEQ multiprobe.

A mixed-method strategy was adopted, combining subjective assessments and objective measurements to provide a comprehensive evaluation of ICU lighting conditions. Objective data were collected using IEQ multiprobe data loggers, while subjective data were obtained from staff through a structured questionnaire. This design, consistent with earlier studies [38,39], enabled a robust assessment by linking physical lighting performance with users’ visual comfort perceptions. Both the online survey and instrumental measurements were conducted in parallel to ensure temporal alignment. Data collection covered two distinct climatic periods, with four weeks of monitoring during the rainy season (10 October–7 November 2023) and four weeks during the dry season (28 May–25 June 2024). This seasonal design allowed for the analysis of potential variations in lighting conditions due to changes in natural daylight availability. For the instrumental measurements, a calibrated IEQ multiprobe device was connected to a laptop computer via a USB port, enabling seamless data acquisition and real-time visualization [40]. The complete setup of the measurement system is shown in Figure 3, and the detailed protocol is provided in Section 2.3.

### 2.2. Subjective Data Acquisition

An online questionnaire was used to collect data on illuminance perception and basic demographic information from hospital employees working in the selected ICUs. To minimize potential bias and ensure confidentiality, there was no direct contact between the researcher and respondents; participation was voluntary and conducted in publicly accessible areas. All participants were informed of the study’s purpose, the intended use of their responses, and their right to privacy before providing consent. Eligible participants included nurses, physicians, and allied health professionals with at least one year of continuous working experience in the ICU during the study period. Patients were excluded to avoid potential bias related to health conditions. The questionnaire was distributed via institutional email to eligible staff, who were encouraged to share the link with colleagues meeting the inclusion criteria. Respondents were asked to provide demographic details, indicate their length of ICU employment, and confirm that their answers reflected actual lighting conditions in the wards. The survey was designed and hosted on the Encuestafacil platform, available in both French and English. Developed in accordance with ISO 7730:2005 [41], the instrument comprised two sections: (1) demographic information (gender, age, professional role, and work experience) and (2) lighting satisfaction parameters, including light intensity, color temperature, flicker, glare, daylight availability, and perceived visual disturbances. Satisfaction was assessed on a five-point Likert scale (1 = very low, 5 = very high), while the prevalence of visual discomfort symptoms was measured on a frequency scale (Never, Rarely, Sometimes, Often, Always). Mean values were calculated as unweighted arithmetic means and reported on the original 1–5 scale or converted to a 0–100 scale for comparative purposes. The survey was conducted in parallel with on-site illuminance measurements to ensure temporal alignment. Ethical approval for the study was obtained from the University Official of Mbuji-Mayi, approval No. UOM-CE/2023/870, dated 13 March 2023, and all procedures were carried out in accordance with the Declaration of Helsinki. Informed consent was obtained electronically from all participants before data collection. A total of 348 healthcare professionals participated: 164 from JSH and 184 from HDC. At HDC, 59.2% of respondents were female and 40.8% were male; at JSH, 54.9% were female and 45.1% were male. Participants in both hospitals included nurses, physicians, and allied health professionals (Table 1).

### 2.3. Quantitative Data Acquisition

Objective illuminance measurements were conducted using a calibrated IEQ multiprobe data logger, connected to a laptop for real-time acquisition and visualization (Figure 2). Before deployment, the device was calibrated according to standardized procedures, with illuminance specifically validated against the Brüel & Kjær 1212 Thermal Comfort Meter to ensure compliance with ISO 8995-1:2002 and CIE guidelines [24] as shown in Figure 3. In each ICU, measurements were taken at three strategically selected fixed points to represent both patient areas and staff work zones.

Probes were placed at a height of 1 m above the floor, corresponding to the typical visual field of seated occupants, and were located at least 3.5 m away from walls and windows to minimize boundary effects and avoid interference from direct sunlight. Data were continuously recorded at 1 min intervals over 24 h cycles, generating high-resolution datasets that captured both daytime and nighttime illuminance conditions across the two climatic seasons. The monitoring specifically targeted horizontal illuminance, which is widely recognized as a primary indicator of lighting adequacy in clinical environments where precision and visual comfort are critical [3,4]. Recorded values were compared against the recommended thresholds outlined in EN 12464-1 for healthcare workplaces [35]. This protocol ensured measurement reliability and provided comprehensive data without disrupting ICU operations or interfering with medical equipment.

### 2.4. Conceptual Framework

**Figure 4:**
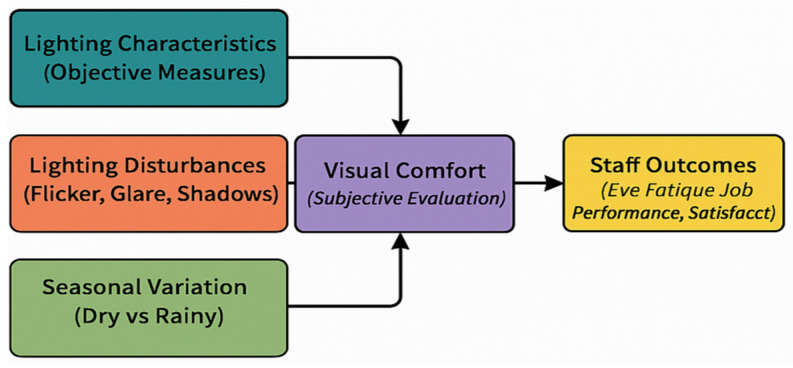
Conceptual framework illustrating the relationships between objective lighting conditions, environmental disturbances, seasonal variation, perceived visual comfort, and staff outcomes in ICUs in the DRC.

The conceptual framework shown in Figure 4 maps the directional relationships between measured lighting conditions, perceived visual comfort, and the resulting occupational outcomes for ICU staff.

Figure 4 presents the conceptual framework developed for this study, illustrating the relationships between key lighting-related factors, perceived visual comfort, and resulting staff outcomes in the ICU environment. Three primary determinants are considered: (i) *Lighting Characteristics* (objective measures such as illuminance, distribution, and color temperature), (ii) *Lighting Disturbances* (flicker, glare, and shadows), and (iii) *Seasonal Variation* (differences between dry and rainy periods). The arrows indicate the hypothesized directional influence of each factor on Visual Comfort (subjective evaluation by healthcare staff). In turn, visual comfort is shown to directly influence Staff Outcomes, which encompass eye fatigue, posture, job performance, and overall satisfaction. By integrating both objective and subjective dimensions, this framework highlights the pathways through which environmental lighting conditions can impact occupational well-being and productivity, thereby providing a structured basis for interpreting the study’s findings.

### 2.5. Statistical Analysis

The quantitative dataset was examined through summary statistics (means, standard deviations, and ranges) and appropriate inferential analyses tailored to the study objectives. One-way Analysis of Variance (ANOVA) was used to examine differences in lighting-related outcomes by hospital and season. Spearman’s correlation coefficients were calculated to assess the relationships between lighting variables and occupational outcomes. A contingency coefficient test was also performed to assess discrepancies between perceived and measured illuminance levels. Statistical analyses were conducted using SPSS v27 (International Business Machines—IBM, NY, USA), with significance thresholds set at *p* < 0.05.

## 3. Results

The results from the comprehensive subjective and objective light quality assessment campaigns are presented below. These findings provide detailed insights into the visual comfort within the ICUs. Advanced statistical tools were used to explore correlations between various light quality parameters. The table below summarizes the objective measurements of illuminance levels in two hospitals, comparing them against established lighting standards, as detailed in Table 2.

Table 2 presents the measured illuminance levels in the ICU of HDC and JSH, compared against the standard illuminance level of 300 lux recommended by the EN 12464-1 standard for workplace lighting. The data reveal that both hospitals significantly underperform in terms of lighting. At HDC, the illuminance levels range from 56.67 lux to 90.00 lux, with a mean value of 83.40 lux. This is considerably below the 300 lux standard, indicating that the lighting is insufficient to support the demanding visual tasks that ICU staff must perform, such as patient monitoring and administering care. Even more concerning is the situation at JSH, where the illuminance levels are lower still, ranging from 46.67 lux to 73.33 lux, with a mean of 67.73 lux. This further deviation from the standard suggests that the lighting conditions in JSH may be inadequate, likely exacerbating the difficulties faced by healthcare staff in performing their duties effectively and safely. The implications of these findings are significant. Adequate lighting is crucial in an ICU setting to ensure that healthcare professionals can perform tasks that require high visual acuity without experiencing excessive eye strain or fatigue. The low illuminance levels reported in both hospitals suggest that the current lighting is not only suboptimal but potentially hazardous, increasing the risk of errors and reducing the overall quality of care. In particular, the even lower levels at JSH might lead to greater visual discomfort and higher levels of job dissatisfaction among the staff, potentially affecting patient outcomes.

### 3.1. Lighting Characteristics and Disturbances

The evaluation of lighting characteristics within the ICUs revealed notable variations in the quality and appropriateness of light sources, color temperature, and daylight availability. These factors are crucial, as they directly impact the visual comfort and overall well-being of healthcare staff, influencing their job performance and satisfaction. Additionally, common disturbances such as flickering and glare were frequently reported, which contributed to visual discomfort and reduced work efficiency. Understanding these elements is crucial for enhancing the working environment in critical care settings. The staff’s ratings of these lighting characteristics and disturbances, as presented in Figure 3, highlight specific areas where the lighting is perceived as inadequate in the hospitals studied.

Table 3 displays the mean ratings (and standard deviations) for the four lighting characteristics and three lighting disturbances. Sixty-five percent of the respondents indicated that at least one of the four lighting characteristics was inappropriate (e.g., very low or low on the scale). The mean scores indicate that four lighting characteristics are appropriate. At HDC Hospital, lighting satisfaction is generally lower, with light sources averaging 20 (SD 18.6), reflecting diverse opinions among patients. Daylight access is even less satisfactory, with an average rating of 14.5 (SD 10.6). Moreover, significant issues such as flickering (mean 19.7, SD 2.1) and glare (mean 18, SD 14.4) indicate a range of discomfort among the occupants. Conversely, JSH experiences a slight improvement in lighting satisfaction, with light sources and color quality averaging 20, albeit with a narrower range of opinions (SDs 17.3 and 13.8). Daylight receives a more favorable assessment, with an average rating of 20 (SD 11.7); however, flickering remains a pronounced problem, with a mean of 24.3 (SD 13.3).

### 3.2. Correlation Between Lighting Variables and Staff Outcomes

Statistical analysis revealed several meaningful correlations between lighting conditions and occupational outcomes in both ICUs. Table 4 presents the correlation coefficients (R-values) between key lighting variables such as type of light source, light level, and flickering and staff outcomes, including satisfaction, job performance, visual fatigue, and posture adjustment.

Table 4 shows that HDC and lighting variables showed strong correlations with staff well-being indicators. Specifically, the type of light source and light level were significantly associated with eye fatigue (r = 0.919 and r = 0.910, respectively). Flickering lights correlated with both eye strain and posture adjustment, indicating visual discomfort during work. Conversely, job performance showed only a weak correlation with light level. Job satisfaction was strongly influenced by job category (r = 0.883), implying variation in lighting needs among staff roles. At JSH, similar trends were observed, although correlations were slightly weaker. Light level was significantly correlated with job performance (r = 0.799), indicating a more direct impact on work efficiency. Flickering also had a strong relationship with posture discomfort (r = 0.889). However, job category had minimal influence on satisfaction at this site, and the association between light level and satisfaction was negligible.

### 3.3. The Impacts on Job Performance and Health

This chart presents ICU staff satisfaction with visual comfort at HDC and JSH during the rainy and dry seasons. The distribution of responses highlights how seasonal variations in daylight availability influence perceptions of lighting adequacy, with implications for both work performance and staff well-being.

**Figure 5:**
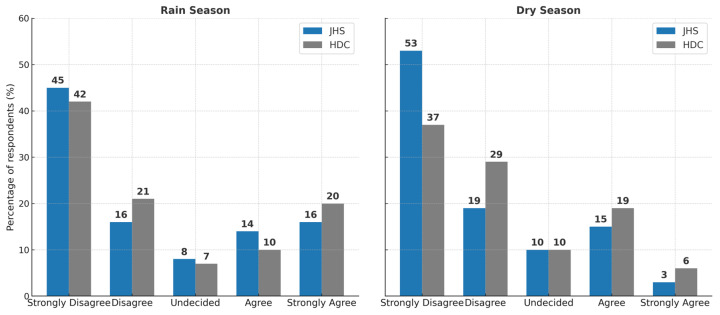
Hospital professionals’ satisfaction with visual comfort during the rainy and dry seasons.

Figure 5 shows clear seasonal differences in satisfaction between the two hospitals. At JSH, dissatisfaction is most pronounced during the dry season, with “Strongly Disagree” responses increasing by eight percentage points and “Strongly Agree” responses falling sharply by thirteen points compared to the rainy season. Moderate increases are also observed in the “Disagree” and “Undecided” categories, while “Agree” responses rise only marginally. This pattern suggests that staff at JSH are highly dependent on natural daylight for visual comfort and that the artificial lighting system is inadequate to maintain high satisfaction levels throughout the year. At HDC, the seasonal effect is less severe but still notable. “Strongly Disagree” responses decrease slightly in the dry season, while “Agree” responses increase by nine points, indicating a modest improvement in moderate satisfaction. However, “Strongly Agree” ratings drop by fourteen points, signaling a significant reduction in the highest satisfaction category. In both hospitals, the decline in top satisfaction ratings during the dry season points to deficiencies in lighting systems, particularly in their ability to deliver consistent, high-quality illumination under varying daylight conditions. These findings show the need for targeted lighting improvements to ensure stable visual comfort and optimal working conditions in ICU environments throughout the year.

### 3.4. Health Symptoms

The survey also assessed the occurrence of common health symptoms potentially associated with IEQ factors. Four symptoms were considered: headaches, eye strain, fatigue, and slipping. Responses were recorded on a five-point Likert scale (Never, Rarely, Sometimes, Often, Always), and prevalence was calculated as the proportion of respondents reporting each symptom at least “Sometimes,” allowing direct comparison between the two hospitals.

**Figure 6:**
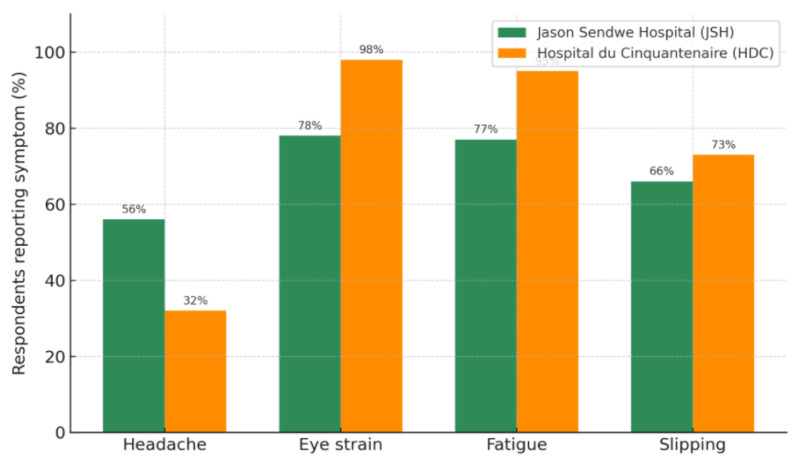
Health symptoms experienced by hospital professionals.

Figure 6 presents the comparative prevalence of four occupational symptoms—headache, eye strain, fatigue, and slipping—among ICU staff at JSH (green bars) and HDC (orange bars). The paired-bar format enables direct visual comparison between facilities. Higher self-reported rates of eye strain and fatigue at HDC align with the objective lighting assessments, which indicated suboptimal illuminance uniformity, elevated glare indices, and measurable flicker from installed luminaires. These lighting deficiencies are recognized risk factors for visual discomfort, asthenopia, and reduced task efficiency, particularly in environments with prolonged near-visual demands and critical care monitoring tasks. Conversely, headaches were more prevalent at JSH, which may be attributable to differences in spectral power distribution, luminaire placement relative to workstations, or occupational stressors unrelated to lighting, such as workflow organization and workload intensity. Slipping incidents were reported at high levels in both hospitals, with a marginally greater frequency at HDC. Floor surface properties may influence such events, cleaning regimes, and suboptimal luminance contrast between walking surfaces and surrounding areas, which can be potentially exacerbated by inadequate horizontal illuminance. The observed patterns in symptom distribution suggest that environmental parameters, especially photometric quality, glare control, and spatial lighting uniformity, can contribute to occupational health outcomes. These findings support the implementation of targeted lighting upgrades and environmental hazard mitigation as part of a comprehensive strategy to enhance ICU working conditions in resource-constrained sub-Saharan African healthcare settings.

### 3.5. Statistical Differences by Hospital and Season

To determine whether differences in lighting-related outcomes were statistically significant between hospitals and across seasons, a two-way ANOVA was performed with Hospital (HDC vs. JSH) and Season (dry vs. rainy) as fixed factors. Four dependent variables were analyzed: lighting satisfaction, job performance, visual fatigue, and posture adjustment. Model assumptions were checked, and effect sizes were expressed as partial η^2^. The results are summarized in Table 5.

Table 5 presents the two-way ANOVA results, showing that both hospital setting and season significantly influenced lighting-related outcomes (Table 5). Lighting satisfaction differed by hospital (F = 5.78, *p* < 0.05, partial η^2^ = 0.03), with staff at JSH reporting higher satisfaction than those at HDC. Visual fatigue also varied significantly by hospital (F = 6.23, *p* < 0.05, partial η^2^ = 0.03), with greater eye strain reported at HDC, consistent with the objective findings of poor illuminance uniformity and glare. In contrast, job performance was strongly affected by season (F = 8.45, *p* < 0.01, partial η^2^ = 0.04), with lower ratings in the rainy season, likely due to the reduced daylight and reliance on inconsistent artificial lighting. Similarly, posture adjustment was more frequently reported during the rainy season (F = 7.90, *p* < 0.01, partial η^2^ = 0.04), indicating greater physical strain under suboptimal lighting conditions. No significant interaction between hospital and season was observed, suggesting that seasonal effects were broadly consistent across both hospitals.

## 4. Discussion

This study found a high correlation between lighting levels and employee satisfaction, job performance, and eye fatigue. A study by Verceles et al. [42], noted low light levels in a medical ICU but found no significant link between these levels and patient outcomes. An intriguing observation from this study was that when the actual illuminance levels were compared with the employees’ perception of Illuminance, in most cases, the employees’ assessment accurately reflected the actual situation. This means there was no substantial underestimation or overestimation between subjective and objective assessments of Illuminance. This finding contrasts with the results of a study by Moore [43], where no relationship was found between individuals’ perceptions of illumination and the actual levels of illumination. Defining objective criteria for optimal lighting can be challenging due to the complexities of lighting situations. About two-thirds of the respondents in the current study felt that at least one of the four evaluated lighting characteristics (light level, light source type, light color, and daylight usage) was not ideal, with light color receiving the lowest ratings. The influence of light color on mood and cognitive performance has been supported by research from Knez and Kers [44]. Other lighting characteristics surveyed in this study, including the type of light source and color, were strongly correlated with employee satisfaction. Over half of the respondents reported significant disturbances due to lighting issues, particularly flickering lights. This highlights the importance of combining subjective assessments and objective measurements when evaluating lighting conditions, especially when objective measurements are challenging or unavailable. The current study’s dual-method approach provides a comprehensive view of lighting conditions by combining the precision of technical measurements with the practical insights gained from staff perceptions.

Subjective evaluations indicated that around two-thirds of respondents, especially those in surgical, pediatric, and general medical wards, were dissatisfied with their lighting conditions. This dissatisfaction aligned with over half of the workstations that did not meet the recommended illuminance levels, suggesting that employee satisfaction is a reliable indicator of actual working conditions. However, a smaller percentage of respondents believed that poor lighting had a negative impact on their job performance. Improving lighting has enhanced job performance, as evidenced by a study among electronic assembly workers, which demonstrated increased production speed with higher illuminance levels. Appropriate lighting is crucial for surgical performance in medical settings, particularly operating rooms. More than half of the respondents reported eye tiredness due to lighting conditions, and about half needed to adjust their posture for better visibility, emphasizing ergonomic concerns. The severity of these issues was more pronounced in surgical, pediatric, and general medical wards than in nursing stations and administrative offices. This aligns with findings from other research, emphasizing the ergonomic importance of proper lighting [45,46]. This study highlights discrepancies between lighting conditions that technically meet standards and those perceived as inadequate by healthcare workers. This insight is crucial for developing interventions that address both technical compliance and user satisfaction.

When asked about potential lighting improvements, respondents favored better maintenance or installation of lighting fixtures and a more appropriate combination of light colors, indicating a preference for holistic lighting solutions that encompass both functional and aesthetic aspects. Adequate lighting is critical to enhancing employee performance, health, safety, and productivity in various work settings [47]. Surprisingly, limited attention has been devoted to environmental factors, particularly lighting, in healthcare settings such as hospitals, where lighting conditions significantly impact staff and patient outcomes. One possible explanation for this discrepancy could be that different lighting levels in a working environment may affect employees’ perceptions. When adequate lighting for task visibility is generally available, employees’ perceptions may not necessarily mirror the illuminance levels. However, when the lighting level falls below recommended standards, employees’ perceptions tend to be more accurate and closely align with the actual illuminance levels. Nevertheless, this hypothesis warrants further investigation to provide a more comprehensive understanding of this phenomenon. It is recognized that establishing objective criteria for defining good lighting can be challenging due to the inherent complexity of any lighting situation. Therefore, it is valuable to consider qualitative aspects of lighting when assessing its quality and impact on individuals [48].

The findings demonstrated a strong correlation between the illumination level and employee satisfaction, job performance, and eye fatigue. These results emphasize the importance of incorporating subjective assessments alongside objective illuminance measurements when evaluating lighting conditions in work environments. Such subjective assessments provide specific and valuable insights into the nuances of lighting conditions that objective metrics may not, especially when objective measurements are impractical or unavailable [48]. The assessed employee satisfaction with lighting conditions through subjective evaluations is the only means to gauge employees’ feelings about their work environment and improve our understanding of those conditions. Alarmingly, approximately two-thirds of respondents working in ICUs expressed dissatisfaction with their lighting environment. This dissatisfaction aligns with the failure to meet recommended illuminance levels in over half of the workstations, suggesting that employee satisfaction accurately reflects the working conditions. This observation echoes findings from previous studies among industrial workers, such as those conducted by Dawal [49].

In contrast to its impact on satisfaction, a smaller proportion of respondents (approximately 26%) believed that lighting had a negative effect on their job performance. Nevertheless, evidence suggests that improving lighting can enhance job performance. For example, a study involving electronic assembly workers reported a 3% increase in production speed when illuminance levels were raised from 800 lux to 1200 lux [50]. While no significant effect on error rates was observed, the study underscores the potential performance benefits of improved lighting. These results highlight the importance of providing suitable lighting in hospitals and healthcare settings to improve employee performance and, potentially, patient outcomes. Regarding health and safety concerns, more than half of the respondents reported experiencing eye fatigue due to the lighting conditions in their working environment, consistent with prior research [51]. Additionally, approximately half of the participants reported needing to adjust their body posture for better visibility due to low light levels. This issue was particularly pronounced in ICU wards, reflecting the challenges faced by healthcare professionals. These findings align with the work performed in the German hospitals, where Ulrich noted that surgeons often struggled with inadequate illumination in operating rooms [52].

This study assessed the lighting conditions in a hospital setting and investigated their impact on employee satisfaction, job performance, and health. Additionally, the study aimed to identify the most suitable methods for improving this workplace’s lighting environment. The study’s primary findings revealed that illumination levels in the surveyed ICU fell below recommended standards. Importantly, employees’ assessments are generally aligned with the actual conditions of their working environment. Furthermore, the study’s correlation coefficients revealed that different light levels had a significant impact on employee satisfaction, job performance, safety, and health. These findings emphasize the importance of considering environmental ergonomics in hospital design to create safer healthcare facilities.

From an ergonomic perspective, addressing posture-related issues is crucial, as awkward postures are known risk factors for musculoskeletal problems among hospital workers, as highlighted by Wadman [53]. Interestingly, lighting conditions greatly impacted falls or slips, with only 64% of respondents reporting such problems. When asked to rate potential improvements to lighting in their working environment, respondents favored better maintenance and installation of lighting fixtures, as well as a more suitable combination of light colors. In contrast, the provision of additional artificial light sources received the lowest rating. This preference is consistent with anecdotal evidence suggesting poorly maintained or installed lighting fixtures can lead to deteriorating visual conditions. Importantly, maintenance applies to electric lighting installations and the cleanliness of internal building surfaces and windows, which can act as light reflectors. Notably, one of the key findings of this study was that measured illuminance levels in ICUs were below the proposed standards [35]. Inadequate lighting in these areas can significantly affect the quality of care and patient safety. In contrast, different hospital areas may have varying lighting requirements, and balancing patient and staff needs is a complex challenge [30]. This study highlights the urgent need for increased attention to lighting in hospital settings, particularly in recognizing the potentially severe consequences of inadequate lighting in ICUs. Striking the right balance between meeting the lighting needs of both patients and staff remains a complex challenge. It emphasizes the pivotal role of environmental ergonomics as a valuable tool in hospital design, emphasizing the imperative of creating safer healthcare facilities through carefully planned lighting solutions. While general lighting is essential to provide adequate illumination for medical procedures and create a pleasant environment conducive to patient recovery, it is worth noting that focusing on task-specific lighting may yield benefits such as improved task visibility, enhanced work focus, and energy conservation advantages by not uniformly illuminating the entire room unless necessary [54].

Furthermore, it is important to acknowledge that only a limited number of studies have provided quantitative light measurements in tropical healthcare settings. Therefore, caution should be exercised when attempting to generalize the findings regarding lighting levels in this study to other healthcare environments. Each healthcare setting may have unique lighting requirements and challenges that warrant individualized assessment and consideration in future research and design endeavors. Moreover, the adequacy of lighting is defined by the lighting infrastructure, which includes the lighting technology, design, and distribution within the hospital building. Additionally, there is a need for a regular energy supply to ensure the proper functioning of hospitals, including the assurance of adequate lighting. The current study’s findings reveal deficits in visual comfort, which impact both patients and hospital workers, underscoring the need for viable interventions that incorporate IEQ, energy, and sustainability considerations. Simply put, if energy supply is adequate, and the right lighting infrastructures are implemented in hospitals’ buildings of the DRC, the associated risk of poor lighting conditions to patient and hospital workers’ comfort, well-being, and performance will be significantly reduced, necessitating technology, managerial, and regulatory strategies for the healthcare settings in the DRC.

### Mitigation Solutions

A holistic approach to lighting is crucial for enhancing the visual environment in ICUs. The first step is the transition to LED lighting. Replacing traditional fixtures with energy-efficient LEDs not only saves energy but also provides adjustable color temperatures. There is a need to use hospital-grade luminaires and redesign the lighting distribution to ensure adequate illuminance in compliance with standard recommendations for such rooms in the hospital. This ensures consistent and high-quality illumination, which is vital for the comfort of patients and medical staff. Building on this, optimizing natural light plays a crucial role through thoughtfully designed ICU layouts with large windows and skylights, as well as the infusion of natural light. Shades that allow gentle daylight filtration to create a soothing and healing environment. This natural illumination is aesthetically pleasing and beneficial for everyone in the ICU. Integrating these improvements with circadian lighting systems presents a comprehensive solution. These systems mimic the natural progression of daylight, changing the color and intensity of light throughout the day. This alignment with the human body’s circadian rhythms can significantly enhance patients’ sleep patterns and overall health.

Moreover, the introduction of individualized lighting controls at patient beds is a critical aspect. This empowers patients to adjust lighting to their preferences, enhancing their comfort and sense of control during their ICU stay. Finally, to address the needs of the medical staff, adjustable task lighting is provided at workstations. Proper illumination is not just a matter of convenience but a necessity for precise medical procedures, ensuring safety and effectiveness in patient care. This interconnected approach to lighting in ICUs represents a blend of technology, human-centric design, and practical functionality, aiming to improve both patient recovery and the working conditions of medical professionals. Overall, considering the inadequacy of electricity supply in the DRC, particularly for hospitals, there is a need to ensure that the power supply is maintained to keep lighting, air conditioning, and hospital operations functioning fully and continuously.

## 5. Conclusions

The current study, as part of an IEQ assessment campaign in the DRC, examined the impact of hospital lighting on employee satisfaction, performance, and safety, finding that many workstations operated at illumination levels below the recommended levels. The inadequacy of lighting is linked to a decreased quality of work environment, highlighting the importance of tailored lighting solutions in critical areas, such as surgical and pediatric wards, to ensure patient care and safety. The study advocates a balance between general and task-specific lighting to enhance focus and conserve energy, emphasizing environmental ergonomics in hospital design for better safety and efficiency. Because few studies provide quantitative light measurements in healthcare settings, generalizing these findings is difficult. Moreover, fewer studies have combined a quantitative and qualitative approach for hospital ICU indoor lighting survey, as in the current study. This study’s focus on ICU lighting conditions in tropical environments, specifically the DRC, is a notable contribution. Most existing research is centered on temperate climates, so this study fills a significant gap by exploring how tropical conditions influence both natural and artificial lighting needs in healthcare settings.

These deficiencies suggest substantial implications for employee satisfaction, job performance, safety, and overall quality of care, underscoring a pressing need for enhanced lighting solutions tailored to the unique challenges presented by tropical environments, which are prevalent in the DRC and similar contexts. Additionally, the study provides a preliminary basis for policymakers and healthcare administrators to draw immediate attention. Moreover, the inconsistency of energy supply, coupled with the challenges observed during the study regarding power supply from the grid and backup power generators (including fueling, maintenance, and overuse), contributes to ensuring adequate lighting requirements in the study region.

Considering the associated risks of inadequate lighting and negative outcomes in healthcare settings underscores the urgency to develop and implement comprehensive national lighting standards for hospitals. These standards should be tailored to account for the unique environmental and infrastructural challenges of the DRC, such as inconsistent natural light availability, power outages, and the use of aging or insufficient lighting technologies. Moreover, there is a need for policy-driven IEQ interventions, as corroborated by previous studies. Thus, policymakers should prioritize formulating guidelines that specify minimum illumination levels for different areas within healthcare facilities, particularly for critical care environments such as ICUs, surgical wards, and pediatric units. These guidelines should advocate for a balanced approach that integrates general ambient and task-specific lighting to meet the diverse needs of healthcare environments. Moreover, there is a strong case for adopting circadian rhythm-based lighting systems in healthcare facilities. These systems can help align artificial lighting with natural light cycles, potentially improving staff well-being and patient recovery by promoting better sleep patterns, reducing fatigue, and enhancing overall alertness and mood. For healthcare administrators, the study underscores the importance of regular, systematic assessments of lighting conditions within their facilities. Maintenance of existing lighting infrastructure is equally crucial; this includes ensuring that lighting fixtures are clean, functioning correctly, and providing the appropriate illuminance levels. Additionally, administrators should consider upgrading to modern, energy-efficient lighting solutions, such as LED systems, which offer better lighting quality and lower operating costs over time. Implementing these improvements can lead to enhanced employee performance, reduced error rates, and a safer, more comfortable environment for staff and patients.

### Limitations and Future Research

While this study provides valuable insights into the impact of lighting conditions in ICUs on employee satisfaction, performance, and safety, several limitations must be acknowledged. One potential limitation is the risk of bias, particularly selection and response biases. The study relied on voluntary participation, which may have led to selection bias, as those with stronger opinions about lighting conditions might have been more inclined to participate. Broad recruitment strategies were employed to mitigate this, and anonymity was assured to encourage diverse participation. Additionally, response bias is a concern, as the subjective survey relied on self-reported data, which could have led to exaggeration or understatement of experiences. To address this, the survey was carefully designed with positively and negatively framed questions and conducted in a neutral, anonymous online environment to promote honest responses.

Another limitation arises from the variability in work environments across different ICUs and wards, which could have influenced both objective lighting measurements and subjective perceptions. Factors such as room layout, window size, and the presence of medical equipment might have affected the lighting conditions, potentially confounding the results. To account for this, measurements were conducted in multiple wards with varied characteristics, ensuring consistency wherever possible.

Seasonal and temporal variability also present a potential confounding factor. Lighting conditions in hospital wards can vary depending on the time of day and season, especially in tropical environments where natural light availability fluctuates significantly. To mitigate this, the study conducted continuous measurements over three weeks, covering both weekdays and weekends, to capture a representative range of lighting conditions. External factors, such as individual differences in vision, mood, or previous experiences, could have influenced how participants perceived the lighting conditions. Although controlling all such variables is challenging, demographic information and work experience were collected to analyze how these factors might have influenced responses. Additionally, the study’s focus on a single hospital in the DRC may limit the generalizability of the findings, as it may not fully represent the range of lighting conditions in other hospitals, particularly in different geographical or climatic regions. Expanding future research to include multiple hospitals, both public and private, would provide a more comprehensive understanding of lighting challenges across various healthcare settings.

Building on these insights, future research should aim to expand and deepen the understanding of lighting conditions in healthcare settings across the DRC and similar regions. A key priority is to expand the investigation to a broader range of hospitals, including both public and private institutions, and to examine the differences between urban and rural healthcare settings. Such comparative studies could reveal important variations in lighting challenges, leading to more tailored and effective lighting solutions. Longitudinal studies would be particularly valuable in understanding the long-term impacts of lighting improvements on healthcare outcomes. Tracking changes over time in hospitals that implement enhanced lighting systems could provide robust evidence of the benefits of such interventions, including reductions in staff fatigue and burnout, improvements in patient recovery times, and overall enhancements in workplace safety and efficiency. Moreover, there is a need for research focused on cost-effective and sustainable lighting solutions suitable for resource-constrained environments. Studies could explore the viability and effectiveness of low-cost LED lighting systems, solar-powered lighting options, and other innovative technologies that provide reliable illumination without significant infrastructure investments. Such research is crucial in making lighting improvements accessible to a broader range of healthcare facilities, particularly in low-income settings.

Additionally, future studies should investigate the specific impacts of circadian rhythm-based lighting systems in tropical healthcare environments. Given the unique natural light patterns in these regions, research could explore how such systems can be optimized to enhance patient and staff well-being. This includes examining the psychological and physiological effects of circadian-aligned lighting on healthcare workers, as well as the potential benefits for patient outcomes, particularly in terms of recovery rates and overall health.

This study was limited to two large urban hospitals, HDC in Kinshasa and Jason JSH in Lubumbashi. While these sites provided valuable insights into ICU lighting conditions in major urban healthcare facilities in the DRC, they may not fully represent the diversity of conditions across other regions of the country, particularly rural or peri-urban areas. Differences in infrastructure, resource availability, building design, and climatic conditions could result in lighting challenges that differ from those observed in this study. Therefore, caution should be exercised in generalizing the findings to all hospitals in the DRC or Sub-Saharan Africa. Future research should include a more diverse sample of healthcare facilities, covering rural, peri-urban, and urban contexts, to improve representativeness and external validity.

This study assessed ICU lighting conditions in relation to healthcare staff satisfaction, comfort, and self-reported performance. Still, it did not collect direct patient outcome data (e.g., recovery time, complication rates, or length of stay). Including such clinical indicators could have provided a stronger link between environmental conditions and patient health. Future research should integrate patient outcome metrics alongside staff-focused measures to better quantify the full impact of lighting quality in ICU settings.

## Figures and Tables

**Table 1:** Distribution of ICU Staff Characteristics across JSH and HDC.

		JSH, Lubumbashi		HDC, Kinshasa	
Division	Category	Counts	Ration (%)	Counts	Ration (%)
Gender	Male	74	45.1	75	40.8
	Female	90	54.9	109	59.2
	Total	164	100	184	100
Age	Less than 30	32	19.5	39	21.2
	31~40	43	26.2	42	22.8
	41~50	32	19.5	46	25.0
	51~60	40	24.4	36	19.6
	Over 60	17	10.4	21	11.4
	Total	164	100	184	100
Occupation	Nurses	94	57.3	101	54.9
	Physicians	39	23.8	40	21.7
	Allied health professionals	31	18.9	43	23.4
	Total	164	100	184	100
Work experience	<5 years	35	21.3	46	25
	6–10 years	55	33.5	59	32.1
	>16 years	74	45.1	79	42.9
	Total	164	100	184	100

**Table 2:** Illuminance levels in ICU areas compared to the recommended standard.

Area	Measured Illuminance (lux)	Statistical Analysis		Recommended Standard (lux)
	Min–Max	Mean	SD	
HDC	56.67–90.00	83.40	7.33	300
JSH	46.67–73.33	67.73	7.05	300

**Table 3:** Mean ratings of lighting characteristics and disturbances in ICUs by healthcare staff.

HDC	Respondents %					
Lighting appropriateness	Very low	Low	Moderate	High	Very high	Mean rate (SD)
Light sources	45.4	32.2	0	16	6.4	20 (18.6)
Light color	40.2	26.9	16.2	11	5.7	28.8 (7.8)
daylights	32.3	22	7	25.5	13.2	14.5 (10.6)
Flickering	21.2	18.2	4.1	21.0	35.5	19.7 (2.1)
Glare	17	19.0	7.1	26.0	31.0	18 (1.4)
JSH						
Light sources	42.1	35.5	7.0	9.2	6.2	20 (17.3)
Light color	38.4	31.3	12.1	8.1	10.1	20 (13.8)
daylights	21.0	39.3	8.4	21.2	10.1	20 (11.7)
Flickering	16.3	24.2	2.5	43.3	13.7	24.3 (13.3)
Glare	20.2	19.5	6.2	22	32.1	20 (9.2)

**Table 4:** Correlation between lighting variables and outcomes.

Lighting Variable/Outcome	HDC (r, *p*-Value)	JSH (r, *p*-Value)
Light Type vs. Satisfaction	0.196, *p* = 0.31	Not significant
Light Source vs. Eye Fatigue	0.919, *p* = 0.009	0.566, *p* = 0.08
Light Level vs. Eye Fatigue	0.910, *p* = 0.01	0.595, *p* = 0.072
Light Level vs. Job Performance	0.127, *p* = 0.55	0.799, *p* = 0.04
Flicker vs. Eye Fatigue	0.637, *p* = 0.10	0.530, *p* = 0.10
Flicker vs. Posture Adjustment	0.751, *p* = 0.05	0.889, *p* = 0.004
Job Category vs. Satisfaction	0.883, *p* = 0.01	0.044, *p* = 0.68
Light Level vs. Satisfaction	Not provided	0.040, *p* = 0.744

**Table 5:** Two-way ANOVA summary of lighting outcome differences by hospital and season.

Dependent Variable	Factor	F-Value	*p*-Value	Partial η^2^
Lighting Satisfaction	Hospital (HDC vs. JSH)	5.78	<0.05	0.03
Job Performance	Season (Dry vs. Rainy)	8.45	<0.01	0.04
Visual Fatigue	Hospital (HDC vs. JSH)	6.23	<0.05	0.03
Posture Adjustment	Season (Dry vs. Rainy)	7.90	<0.01	0.04

## Data Availability

The data are unavailable due to privacy concerns.
